# Insights into the Biosynthesis of Caffeoylquinic Acids in *Uncaria tomentosa* Root Cultures

**DOI:** 10.3390/plants15203224

**Published:** 2026-10-21

**Authors:** Juliana da Silva Coppede, Lyamara M. O. Pavão, Beatriz de Souza Rodrigues, Ana Maria S. Pereira, Adriana A. Lopes

**Affiliations:** Biotechnology Unit, Universidade de Ribeirão Preto (UNAERP), Ribeirão Preto 14096-900, Brazil; jcoppede@unaerp.br (J.d.S.C.); lyamara.oliveira@sou.unaerp.edu.br (L.M.O.P.); beatriz.drodrigues@sou.unaerp.edu.br (B.d.S.R.); apereira@unaerp.br (A.M.S.P.)

**Keywords:** chlorogenic acid, 3,4-di-O-caffeoylquinic acid, cat’s claw, shikimate pathway

## Abstract

The DNA repair action of *Uncaria tomentosa* has been attributed to chlorogenic acid and 3,4-di-O-caffeoylquinic acid present in the bark and roots of the plant. Extractive harvesting of the species has compromised its survival in natural environments. Thus, establishing in vitro root production and investigating the biosynthetic pathway of these compounds could make their production sustainable. In this context, an in vitro growth curve of *U. tomentosa* roots was established in culture medium supplemented with glucose and 5.37 μM 1-naphthaleneacetic acid. Subsequently, the root culture was grown in 1-^13^C-glucose. Production of labeled chlorogenic acid and 3,4-di-O-caffeoylquinic acid was confirmed by spectroscopic methods, and their contents were determined by HPLC. *U. tomentosa* root cultures produced 2.03 mg·g^−1^ dry weight (DW) of chlorogenic acid at 27 days of cultivation and 3.44 mg·g^−1^ DW of 3,4-di-O-caffeoylquinic acid at day 18. These compounds were isotopically labeled, exhibiting an incorporation pattern consistent with the shikimate pathway. The established *U. tomentosa* root culture represents a promising biotechnological platform for obtaining these compounds.

## 1. Introduction

**Figure 1:**
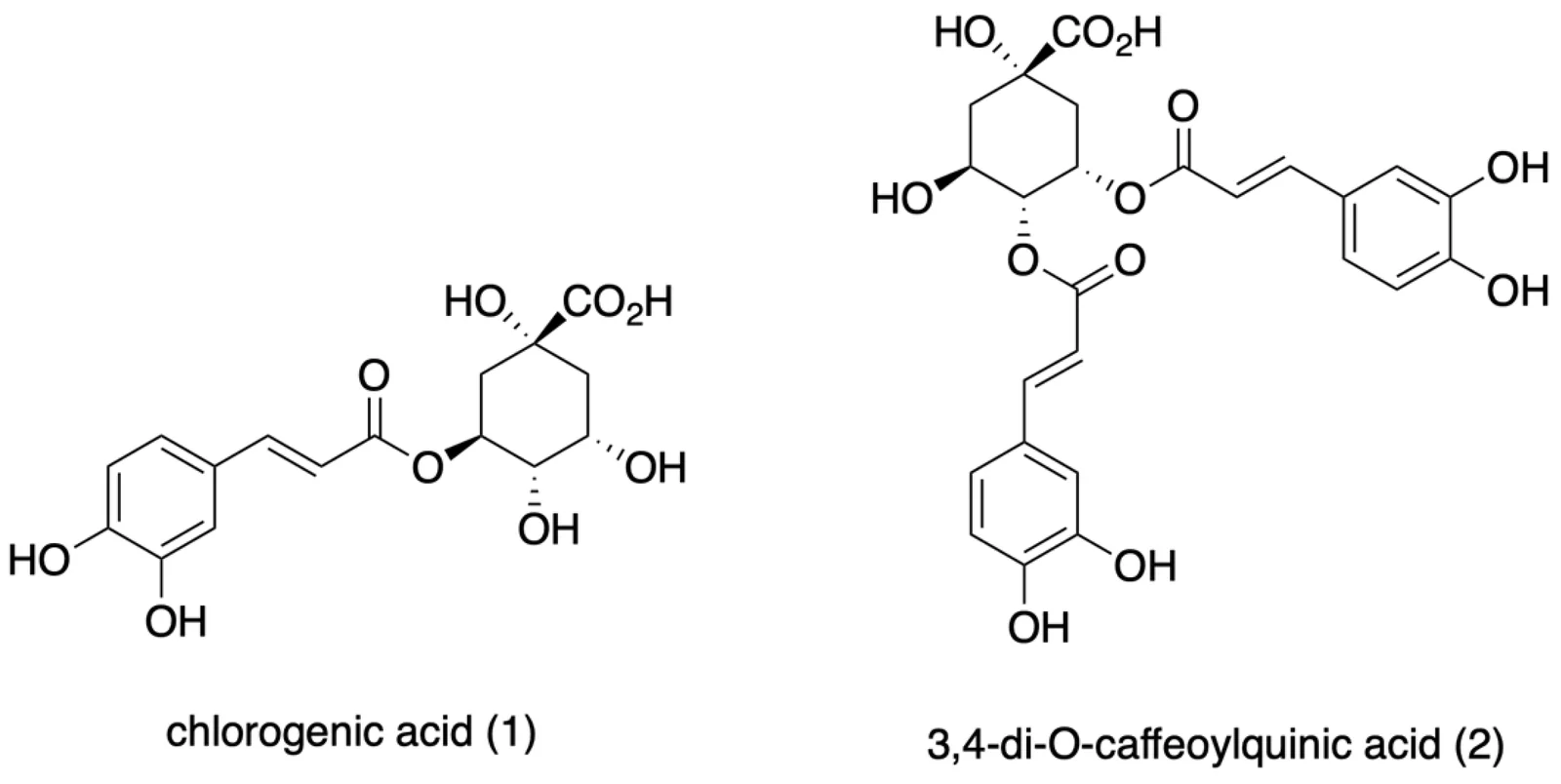
Chemical structures of CQAs identified in *Uncaria* species.

It is well known that herbal teas prepared from medicinal plants provide health benefits, mainly due to their high content of bioactive phenolic compounds [1]. *Uncaria tomentosa* (Willd. ex Schult.) DC. (Rubiaceae) is widely recognized and known worldwide as a botanical drug for its potent anti-inflammatory [2,3,4] and immunomodulatory [5,6,7] activities. Carboxyl alkyl esters (CAEs) or caffeoylquinic acids (CQAs), also known as quinic acid derivatives, such as chlorogenic acid (1) and 3,4-di-O-caffeoylquinic acid (2) (Figure 1), are a class of compounds produced by *Uncaria* species that are well known for their anti-inflammatory properties [8] and DNA repair [9], having attracted increasing attention in recent years.

Chlorogenic acid (1) consists of different isomers and is one of the most abundant phenolic compounds found in medicinal plants, exhibiting multiple pharmacological activities, particularly its antioxidant and anti-inflammatory effects [10]. Studies have demonstrated that compound **1** exhibits multiple biological activities, including antioxidant and anti-inflammatory effects, mediated through the modulation of metabolic homeostasis as well as inflammatory and oxidative responses [11]. Its anti-inflammatory effects are associated with the suppression of inflammatory mediator production and the attenuation of tissue damage through inhibition of NF-κB signaling pathways [12]. Furthermore, beneficial effects have been reported in several pathological conditions, including cardiovascular diseases, diabetes, obesity, neuroprotection, and metabolic disorders [11].

The compound 3,4-di-O-caffeoylquinic acid (2) also exhibits significant pharmacological properties. Structurally, the presence of a greater number of conjugated double bonds in the caffeoyl moieties enhances radical stabilization through resonance, thereby enhancing its antioxidant activity [13]. In human keratinocytes, protection against environmental oxidative damage (particularly that induced by air pollution and UVB radiation) was observed; this is associated with the inhibition of pathways triggered by oxidative stress, suggesting a protective effect [14]. Furthermore, compound **2** demonstrated protective effects on vascular endothelial cells and vascular function by reducing oxidative stress and reactive oxygen species through its antioxidant activity [15]. Compound **2** has been shown to exhibit activity against enterovirus A71 by inhibiting viral entry into host cells [16]. Moreover, this compound exerts various pharmacological activities, including antibacterial, anticancer, hypoglycemic, hypotensive, hepatoprotective, and antiviral effects [17].

Phytomedicines derived from *U. tomentosa* are traditionally obtained from the stem bark; however, due to the risk of extinction facing this species [18], biotechnological approaches, such as the cultivation of medicinal plant roots, have attracted increasing interest. Therefore, this study aimed to establish root cultures of *U. tomentosa* to elucidate the biosynthetic pathway involved in the formation of quinic acid derivatives through supplementation with 1-^13^C-glucose.

## 2. Results

**Figure 2:**
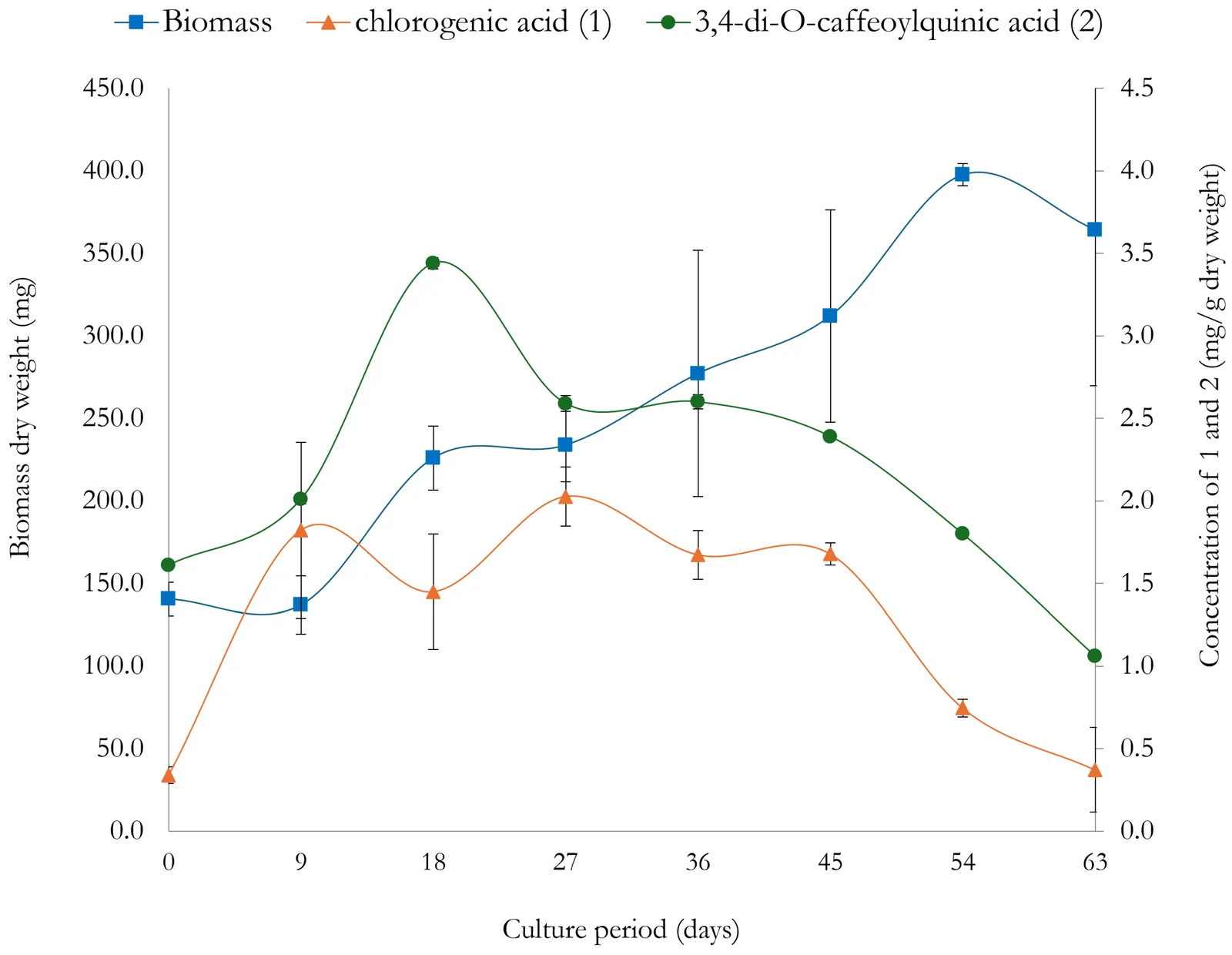
Production of chlorogenic acid (**1**) and 3,4-di-O-caffeoylquinic acid (**2**), along with dry weight accumulation, in *Uncaria tomentosa* root cultures (*n* = 24) maintained for 63 days under a 16 h light/8 h dark photoperiod.

*U. tomentosa* adventitious roots showed that peak biomass production was at day 54 of culture (397.67 mg). HPLC-UV analysis of the crude extract obtained from *U. tomentosa* root cultures revealed the production of 1.82 mg·g^−1^ dry weight (DW) of compound **1**, with peak accumulation observed at 9 days of culture. A second maximum production was observed at day 27 reaching 2.03 mg·g^−1^ DW. Compound **2** also presented accumulation, reaching 3.44 mg·g^−1^ DW at day 18 (Figure 2). Notably, the production of compound **2** was 1.69-fold higher than that of compound **1**.

After isolation of quinic acid derivatives 1 and 2, all ^13^C NMR (nuclear magnetic resonance) data allowed us to establish the chemical structure in comparison with literature data [19,20]. Additionally, the major product ions of compounds **1** and **2** were characterized by ESI-QTOF-MS/MS (Table S1). The deprotonated molecular ions [M − H]^−^ were observed at *m*/*z* 353.0881 for compound **1** and *m*/*z* 515.1186 for compound **2**, corresponding to mass errors of +0.83 and −1.75 ppm, respectively. The MS^1^ and MS^2^ data obtained for compound **1**, as well as the MS^1^, MS^2^, and MS^3^ data obtained for compound **2** (Table S2, Figures S1–S4), were consistent with those previously reported by [21]. Complementary NMR data (^1^H, ^13^C, HSQC, and HMBC) supported the structural assignment of compound **2** (Figures S5–S8, Table S3) in agreement with previously reported data [20].

**Figure 3:**
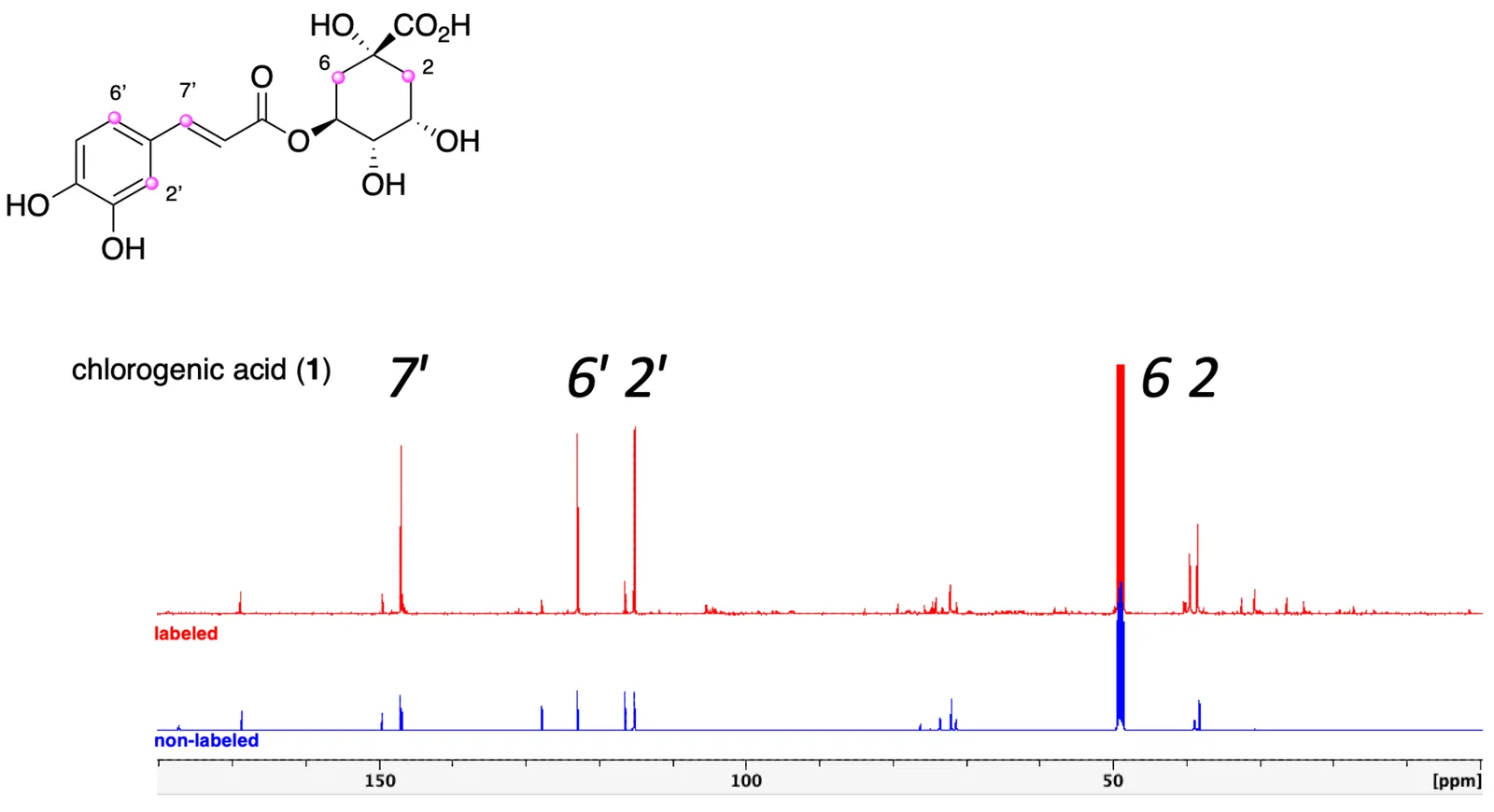
^13^C NMR (150 MHz, CD_3_OD) spectra of chlorogenic acid labeled and unlabeled isolated from *U. tomentosa* root cultures following metabolism of 1-^13^C-glucose and glucose, respectively.

**Figure 4:**
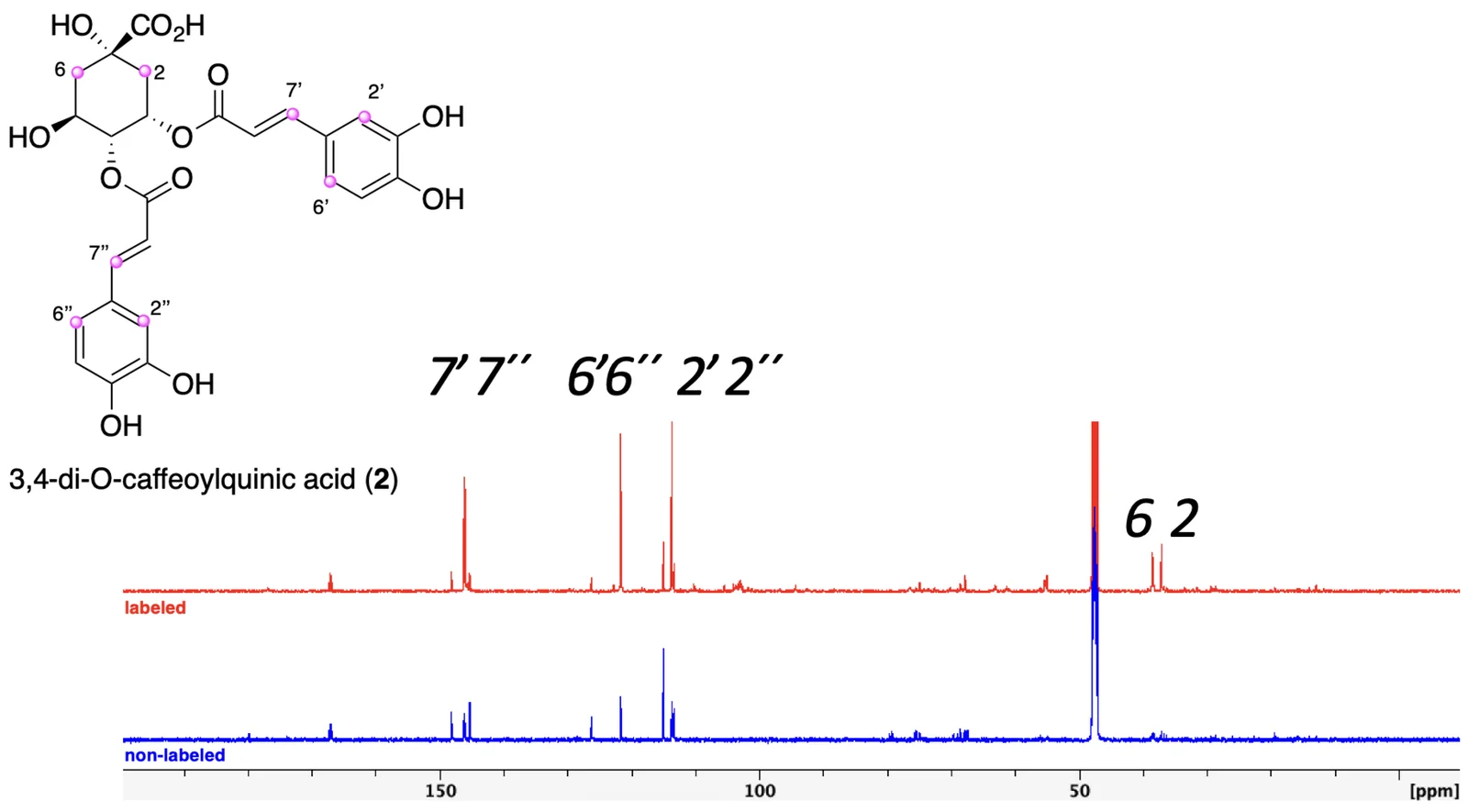
^13^C NMR (150 MHz, CD_3_OD) spectra of 3,4-di-O-caffeoylquinic acid labeled and unlabeled isolated from *U. tomentosa* root cultures following metabolism of 1-^13^C-glucose and glucose, respectively.

Feeding experiments revealed ^13^C enrichment by comparing the relative intensities of labeled and unlabeled signals for both compounds. Incorporation of ^13^C from 1-^13^C-glucose into compound **1** was observed at C-2 (δ 38.2), C-6 (δ 38.3), C-2′ (δ 116.5), C-6′ (δ 122.9), and C-7′ (δ 146.8). The ^13^C NMR spectrum from 2 showed incorporation of ^13^C at C-2 (δ 38.6), C-6 (δ 39.9), C-2′/2″ (δ 115.1), C-6′/6″ (δ 123.1), and C-7′/7″ (δ 147.6) (Figure 3 and Figure 4). Incorporation patterns of CQAs were determined by quantitative ^13^C NMR comparing the relative intensities of the labeled and non-labeled CQAs signals for 1 and 2 (Tables S4 and S5). For compound **1**, the lowest estimated ^13^C abundance was observed at C-7′ (4.1%), whereas the highest estimated ^13^C abundance was detected at C-2′ (6.2%). For compound **2**, the lowest estimated ^13^C abundance was observed at C-2 (4.4%), while the highest estimated ^13^C abundance was detected at C-2′/C-2″ (5.9%). Our findings contribute to the current understanding of the stepwise biosynthesis of quinic acid derivatives by elucidating the incorporation of a ^13^C-labeled precursor, therefore supporting the biosynthetic route. Thus, this study provides evidence supporting that compounds **1** and **2** are biosynthesized via the shikimate pathway (Figure S9).

## 3. Discussion

Earlier studies on *U. tomentosa* root cultures reported that the maximum biomass accumulation occurs at 30 days; however, these findings were obtained under conditions lacking plant growth regulators in the culture medium [22]. A more recent study showed the maximum biomass production on the 35th day of cultivation, coinciding with the production of compound **1** (58.47 mg·g^−1^ dry weight), and compound **2** also showed an accumulation of 20.28 mg·g^−1^ dry mass; however, the optimal day and the time of the greatest accumulation for compound **2** observed by [23] was the 42nd day of cultivation, when the content reached 32.53 mg·g^−1^ dry mass [23]. In the present study, the maximum concentrations of compounds **1** and **2** were 2.03 and 3.44 mg·g^−1^ dry weight. A comparison of the production profiles revealed differences in the timing of metabolite accumulation, with peak production of compound **1** occurring at 27 days and that of compound **2** at 18 days. The temporal profiles revealed a marked uncoupling between biomass accumulation and caffeoylquinic-acid accumulation. 3,4-Di-O-caffeoylquinic acid reached its maximum concentration at day 18 and chlorogenic acid at day 27, whereas root biomass continued to increase until day 54. Thus, harvesting at the maximum biomass would not maximize the concentration of the target metabolites. The decline in caffeoylquinic acids during later culture stages may reflect changes in developmental metabolism, dilution caused by continued biomass accumulation, metabolic turnover or conversion, sequestration, or a reduction in biosynthetic flux as cultures approach the stationary/senescent phase. These results indicate that the optimal harvest window for metabolite recovery precedes the maximum biomass accumulation and that production conditions require further optimization.

The lower metabolite concentrations observed in the present study, compared with those reported by [23], cannot be attributed to a single cultivation factor. Notably, the two studies employed *U. tomentosa* accessions of distinct geographical origins within the Brazilian Amazon. The plant material used in the present study originated from Bannach, Pará State, whereas [23] investigated an accession from Tarauacá, Acre State. These differences in geographical origin may be associated with genetic and physiological variability among accessions, potentially contributing to the observed differences in secondary metabolite accumulation.

Furthermore, differences in carbon source may contribute to the altered accumulation profile; however, other differences in culture conditions, inoculum physiology, growth-regulator regime, and harvest timing may also influence caffeoylquinic-acid accumulation. Because these variables were not independently tested, the present data do not establish the cause of the lower productivity.

As shown in Figure 2, the reduction observed in metabolite levels and biomass at 63 days of culture may be associated with nutrient depletion in the culture medium, physiological limitations of the system, or the onset of root senescence processes [24]. Cultures maintained for prolonged periods frequently exhibit reduced biosynthetic activity due to metabolite accumulation, limited carbon availability, and physiological alterations associated with the stationary growth phase [25].

The biosynthesis of this class of compounds is compartmentalized. In the initial steps of the shikimate pathway, erythrose-4-phosphate reacts with phosphoenolpyruvate (PEP) in plastids (Figure S9), leading to the formation of the intermediate 3-dehydroquinic acid [26]. This intermediate undergoes a reversible reduction to produce quinic acid, while, following dehydration and reduction, it is converted into shikimic acid, the main branching point for phenylalanine biosynthesis. Shikimic acid, upon reaction with a second molecule of PEP, gives rise to chorismic acid, which subsequently leads to phenylalanine formation [27].

Subsequently, phenylalanine is transported from the plastid to the cytosol via specific plastidial transporters [26]. Other metabolites, such as quinic acid, shikimic acid, and chorismic acid, are also directed to the cytoplasm [28]. In the cytosol, phenylalanine undergoes deamination to form cinnamic acid, a key starting point for phenylpropanoids. This constitutes a key regulatory step, as it regulates the carbon flux available for the synthesis of compound **1**. Subsequently, compound **1** is transported to the vacuole, where it reacts with caffeic acid to form compound **2** [29]. Based on proposed models of phenylpropanoid compartmentation, chlorogenic acid may undergo intracellular transport and may serve as a precursor within subsequent caffeoylquinic-acid metabolism. However, the localization, transport steps, and direct conversion leading to 3,4-di-O-caffeoylquinic acid were not examined in the present study; therefore, this sequence should be regarded as a literature-based hypothesis.

## 4. Materials and Methods

### 4.1. Plant Material

The *U. tomentosa* plant material used in this study originated from Bannach, Pará State, Brazil. The collection was previously registered in Sistema Nacional de Gestão do Patrimônio Genético e do Conhecimento Tradicional Associado (SISGEN), (Process number: A4E1F8C). The specimen was identified by Piero Giuseppe Delprete (Herbier de Guyane, Institut de Recherche pour le Développement, Cayenne, French Guiana), and voucher material was deposited in the Herbarium of the Universidade de Ribeirão Preto (UNAERP) under the accession code HJBOP 3135.

### 4.2. Chemicals

Chlorogenic acid (1), 3,4-di-O-caffeoylquinic acid (2), 1-naphthaleneacetic acid (NAA), and 1-^13^C-glucose were purchased from Sigma-Aldrich^®^ (St. Louis, MO, USA). LC-grade solvents were supplied by J. T. Baker^®^ (Phillipsburg, NJ, USA).

### 4.3. Establishment of Uncaria tomentosa Adventitious Roots

Adventitious root cultures were established from micropropagated *U. tomentosa* plantlets which were already maintained under aseptic conditions; therefore, no additional surface sterilization was required. Roots were excised from the plantlets and transferred to 250 mL Erlenmeyer flasks containing 100 mL of Murashige and Skoog (MS) basal medium [30] supplemented with 1.0% (*w*/*v*) glucose and 5.37 μM NAA, with the pH adjusted to 6.0. These three inputs were purchased from Sigma-Aldrich® (St. Louis, MO, USA). These cultures were subjected to orbital agitation at a rate of 110 rpm and sub-cultivated over a period of three weeks. Adventitious roots were maintained in the growth room, where the temperature was maintained at 25 ± 2 °C, the relative humidity was 55–60%, and the photoperiod was 16 h with an intensity of 40 µmol m^−2^s^−1^, provided by 85 W cool-white General Electric fluorescent lamps.

### 4.4. Determination of the Growth Kinetics of Uncaria tomentosa Adventitious Root Cultures

For growth kinetics experiments, roots (2.00 ± 0.01 g fresh weight) were inoculated into each culture flask. Day 0 corresponded to the baseline immediately after inoculation; roots assigned to this time point were harvested immediately and dried for 24 h at 45 °C, and their dry weight was determined. The experiment followed a completely randomized design with three independent replicates (*n* = 24 culture flasks in total). Samples were harvested at regular intervals from day 0 to day 63 of culture, weighed and dried in a circulating air oven for 24 h at 45 °C. After drying was completed, the DW of the roots was determined, and the samples were stored at −20 °C.

### 4.5. Quantification of Chlorogenic Acid and 3,4-di-O-Caffeoylquinic Acid in Uncaria tomentosa Adventitious Root Cultures

Root samples (n = 3) were extracted with methanol (at a ratio of 100 mg·mL^−1^), and the samples were kept at rest and protected from light for 24 h. After this period, the same proportion of methanol was added again, and the extraction process was repeated under the same conditions. Crude extracts obtained (8 mg·mL^−1^) were prepared in methanol, filtered through a 0.45 μm membrane and injected into a Shimadzu HPLC system consisting of LC-10AD pumps, a DGU-20A3 degasser, an SCL-10 system controller, an SIL-10AD automatic injector, and an SPD-M10A UV/VIS DAD detector (Shimadzu Corporation, Kyoto, Japão) operated by Class VP software. A Kinetex^®^ XB-C18 column (250 mm × 4.6 mm, 5 µm) coupled to the respective pre-column was used. The solvent system consisted of 0.1% acetic acid in water (A) and methanol (B). An initial isocratic elution at 10% B was maintained from 0.01 to 2.01 min, followed by a linear gradient from 10% to 100% B over 2–32 min and a final isocratic step at 100% B from 32 to 40 min. The injection volume was 20 μL, and detection was performed at 330 nm. Quantification was carried out by external standardization using calibration curves of compound **1** (1.0 to 0.0781 mg·mL^−1^) and compound **2** (0.128 to 0.004 mg·mL^−1^). Samples and standards were analyzed in triplicate. The analytical performance of the HPLC-DAD method was evaluated in terms of linearity, precision, accuracy, limit of detection (LOD), and limit of quantification (LOQ). Linearity was assessed by linear regression of the analytical standard calibration curve. Precision was evaluated as repeatability and expressed as relative standard deviation (RSD), whereas accuracy was assessed by recovery experiments at low, medium, and high concentration levels. LOD and LOQ were calculated from the calibration data according to the procedure described above (Table S6, Figure S10).

### 4.6. Feeding Experiments Using 1-^13^C-Glucose in U. tomentosa Adventitious Root Cultures

After establishing *U. tomentosa* adventitious root cultures, the roots (2.0 g each; n = 20) were inoculated in 100 mL of MS basal medium culture [30] in each 250-mL Erlenmeyer flask, with 1-^13^C-glucose at a concentration of 0.5% (*w*/*v*), 5.37 μM of NAA and the pH adjusted to 6.0. Additionally, a control group of roots (2.0 g each; n = 10) was inoculated in 100 mL of MS basal medium culture [30] in each 250 mL Erlenmeyer flask, glucose at a concentration of 0.5% (*w*/*v*), 5.37 μM of NAA and the pH adjusted to 6.0. After 18 days of incubation, roots from the flasks within each experimental condition were pooled, harvested, weighed, and dried in a forced-air oven at 45 °C for 24 h. After drying, the methanolic extract was prepared as described above.

### 4.7. Isolation of ^13^C-Labeled and Unlabeled 1 and 2 by C-18 Solid-Phase Cartridge (SPE)

The unlabeled methanolic extracts (451 mg) and labeled methanolic extracts (644 mg) were subjected to concentration and fractionation using the LCSPE-fast protocol according to the literature [31]. The methanolic extracts were fractionated using Supelclean™ LC-18 solid-phase extraction cartridges (Supelco, Sigma-Aldrich, St. Louis, MO, USA). Firstly, C-18 SPE cartridges were conditioned in 100% MeOH (40 mL) and subsequently conditioned with the appropriate solvent used for sample preparation (100% H_2_O, 40 mL). The sample was dissolved in 100% H_2_O (10 mL) and eluted through the cartridge using four different methanol–H_2_O proportions yielding five fractions: fraction I (20.5% MeOH), fraction II (29% MeOH), fraction III (41% MeOH), fraction IV (58% MeOH), and fraction V (100% MeOH). Additionally, ethyl acetate (40 mL) was used for the final cartridge wash. Fraction II (enriched in compound **1**) and fraction IV (enriched in compound **2**) (labeled and unlabeled) were isolated by semi-preparative HPLC.

### 4.8. Isolation of ^13^C-Labeled and Unlabeled 1 and 2 by Semi-Preparative HPLC

Fraction II (334 mg labeled; 16.5 mg unlabeled) and fraction IV (57 mg labeled; 21.7 mg unlabeled) were subjected to semi-preparative HPLC using Shimadzu^®^ high-performance liquid chromatography, coupled to LC-6AD pumps, a CBM-20A system controller, DGU-20A degasser, SPD-M20A UV/VIS diode array detectors, SIL-10AF automatic injector (Shimadzu Corporation, kyoto, Japão), and Class VP software. For semi-preparative analysis, an Agilent Zorbax^®^ Eclipse XDB-C18 (250 × 10.0 mm, 5 µm particle size) was used. The mobile phase was prepared using 0.1% acetic acid in water (A) and methanol (B), and chromatographic separation was performed using the following elution gradient [32]: 20% B at 0.01 min; 66% B at 30.00 min; 100% B from 30.01 to 32.00 min; and 15% B from 32.01 to 35.00 min. The flow rate was 3.0 mL min^−1^, the injection volume was 100 µL, and detection was performed at λ = 330 nm. A total of 5.0 mg of compound **1** (labeled and unlabeled) and 2.0 mg of compound **2** (labeled and unlabeled) were isolated and subsequently subjected to ^13^C NMR analysis.

### 4.9. NMR Analysis

NMR spectra were recorded on a Bruker Avance III HD 600 MHz spectrometer. ^13^C NMR spectra of ^13^C-labeled and non-labeled isolated 1 and 2 were obtained using CD_3_OD.

### 4.10. UPLC-QTOF/HRMS Analysis

High-resolution mass spectrometry (HRMS) analyses were performed by direct injection using an Agilent^®^ 1290 Infinity II UHPLC system coupled to an Agilent^®^ 6545 QTOF high-resolution mass spectrometer (QTOF/HRMS) (Agilent Technologies, Palo Alto, CA, USA) at the Natural Products Laboratory of the Federal University of São Carlos (UFSCar). The mobile phases consisted of A-H_2_O containing 0.1% formic acid and B-CH_3_CN containing 0.1% formic acid. The gradient program increased from an initial 5% B to 100% B over 14 min. The flow rate was set at 0.3 mL min^−1^, the injection volume was 5 μL, and the column temperature was maintained at 40 °C.

Mass spectra were acquired in negative-ionization mode using Auto MS/MS (Agilent Technologies, Palo Alto, CA, USA). Full-scan MS data were recorded over an *m*/*z* range of 100–1500, whereas MS/MS spectra were acquired over an *m*/*z* range of 70–1500. The eight most intense precursor ions were automatically selected for fragmentation. The instrumental parameters were set as follows: capillary voltage, 3000 V; nozzle voltage, 1000 V; gas temperature, 350 °C; drying gas flow rate, 12 L min^−1^; nebulizer pressure, 35 psi; sheath gas temperature, 300 °C; fragmentor voltage, 110 V; and skimmer voltage, 60 V. MS/MS spectra were acquired using multiple collision energies of 10, 15, 20, and 30 eV. Data acquisition and analysis were performed using Qualitative Navigator software, version B.08.00.

### 4.11. Statistical Analysis

The statistical analysis was performed using the Scott Knott test at the 5% confidence level with the aid of Sisvar software, version 5.3 [33].

## 5. Conclusions

Our results suggest that *U. tomentosa* root cultures represent a promising biotechnological platform for producing chlorogenic acid and 3,4-di-O-caffeoylquinic acid under the cultivation conditions investigated. Furthermore, feeding experiments with 1-^13^C-glucose revealed enrichment patterns consistent with biosynthesis via the shikimate pathway for both compounds. Subcellular compartmentalization and the direct conversion of chlorogenic acid into 3,4-di-O-caffeoylquinic acid remain hypotheses to be tested. The productivity of these compounds still requires optimization. Nevertheless, the system offers a sustainable alternative to the extractive harvesting of bark from this species, which is vulnerable to extinction.

## Data Availability

The data presented in this study are available on request from thecorresponding author, and the data are not publicly available due to privacy restrictions.

## Supplementary Materials

The following supporting information can be downloaded at: https://www.mdpi.com/article/10.3390/plants15203224/s1, Figure S1: Full-scan QTOF/HRMS mass spectrum of compound **1** (m/z 353.0881); Figure S2: MS/MS fragmentation spectrum of compound **1** (m/z 353.0876); Figure S3: Full-scan QTOF/HRMS mass spectrum of compound **2** (m/z 515.1186); Figure S4: MS/MS fragmentation spectrum of compound **2** (m/z 515.1183); Figure S5: ^1^H NMR spectrum of compound **2** (600 MHz, CD_3_OD); Figure S6: ^13^C NMR spectrum of compound **2** (150 MHz, CD_3_OD); Figure S7: HSQC spectrum of compound **2** (150 MHz, CD_3_OD); Figure S8: HMBC spectrum of compound **2** (150 MHz, CD_3_OD); Figure S9: Biosynthesis of caffeoylquinic acids (compounds **1** and **2**); Figure S10: Chromatographic profiles of *Uncaria tomentosa* root extracts obtained from (a) the control experiment; b) the isotope-labeling experiment; (c) chlorogenic acid (1) and (d) 3,4-di-O-caffeoylquinic acid (2) standards; Figure S11: *Uncaria tomentosa* root cultures. Table S1: ESI-QTOF-MS data of compounds **1** and **2**; Table S2: Negative-ion MS^1^, MS^2^ and MS^3^ data of compounds **1** and **2**; Table S3: ^1^H (600 MHz) and ^13^C (150 MHz) NMR spectroscopic data of compound **2** recorded in CD_3_OD; Table S4: ^13^C NMR data of 1 and 1-labeled (CD_3_OD) after incorporation of 1-^13^C-D-glucose into root cultures; Table S5: ^13^C NMR data of 2 and 2-labeled (CD_3_OD) after incorporation of 1-^13^C-D-glucose into root cultures; Table S6: Analytical validation parameters of the HPLC-DAD method for the quantification of compound **2**.
