# Supplementary material for: Insights into the Biosynthesis of Caffeoylquinic Acids in Uncaria tomentosa Root Cultures
Source: Plants (Basel). 2026 Oct 21;15(20):3224. doi: 10.3390/plants15203224 (PMC13709219; doi:10.3390/plants15203224)
Supplement: Supplementary file 1 [file plants-15-03224-s001.zip › plants-4511071-supplementary.pdf]

Supplementary Information

Table S1. ESI-QTof-MS data of compounds 1 a 2.

Compound	Molecular formula	Ion	Calculated *m/z*	Experimental *m/z*	Error (ppm)
1	$C_{16}H_{18}O_9$	$[M–H]^-$	353.0878	353.0881	+0.83
2	$C_{25}H_{24}O_{12}$	$[M–H]^-$	515.1195	515.1186	−1.75

Table S2. Negative ions MS^1, MS^2 and M^3 data of compound 1 and 2.

Compound	MS^1 (m/z)	MS^2 (m/z)	MS^3 (m/z)
1*	353.3	191.5; 179.5; 161.4	127.1; 172.2
1	353.0881	191.0560; 179.0345; 161.0238	-
2*	515.7	353.4;335.6; 173.5	179.5; 191.7; 172.9; 135.6
2	515.1186	353.0876; 335.0759; 173.0450	179.0343; 191.0554; 135.044

*Literature data (Clifford et al. 2003).

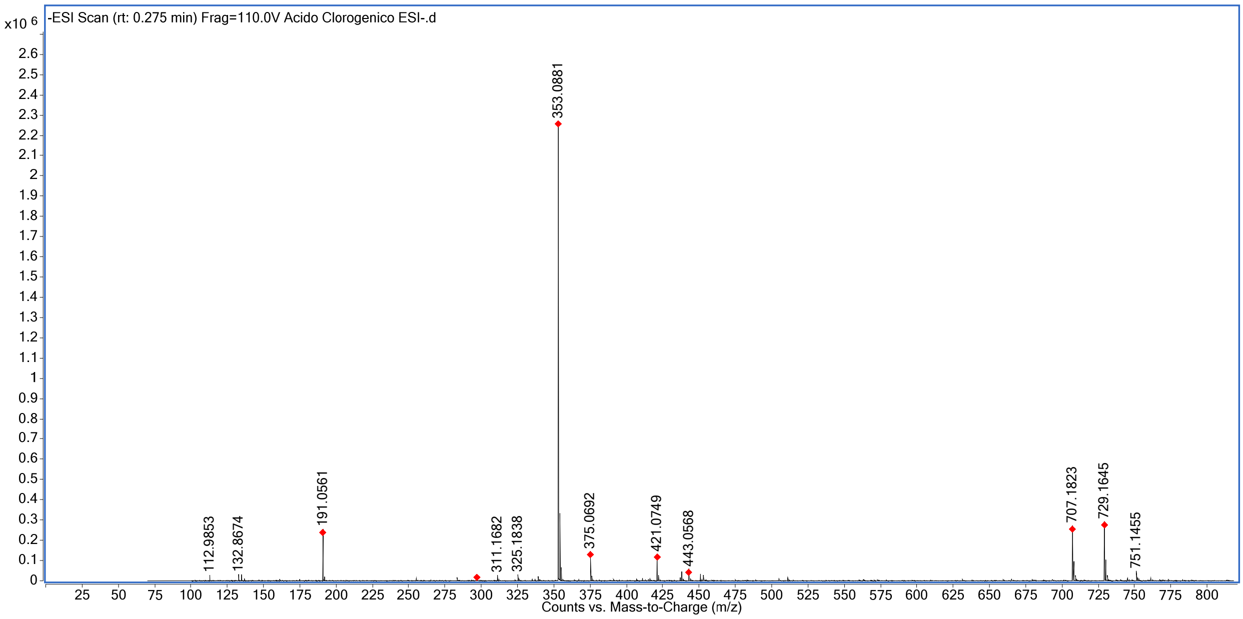

Figure S1. Full-scan QTOF/HRMS mass spectrum of 1 (m/z=353.0881).

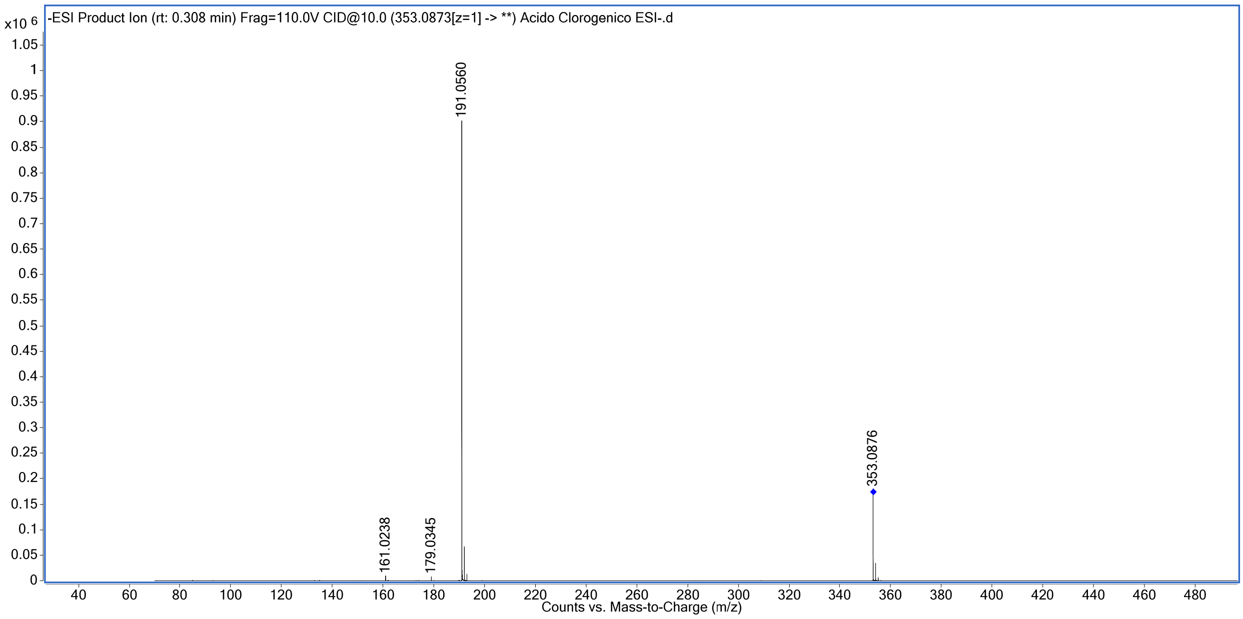

Figure S2. MS/MS fragmentation spectrum of compound 1 (m/z=353.0876).

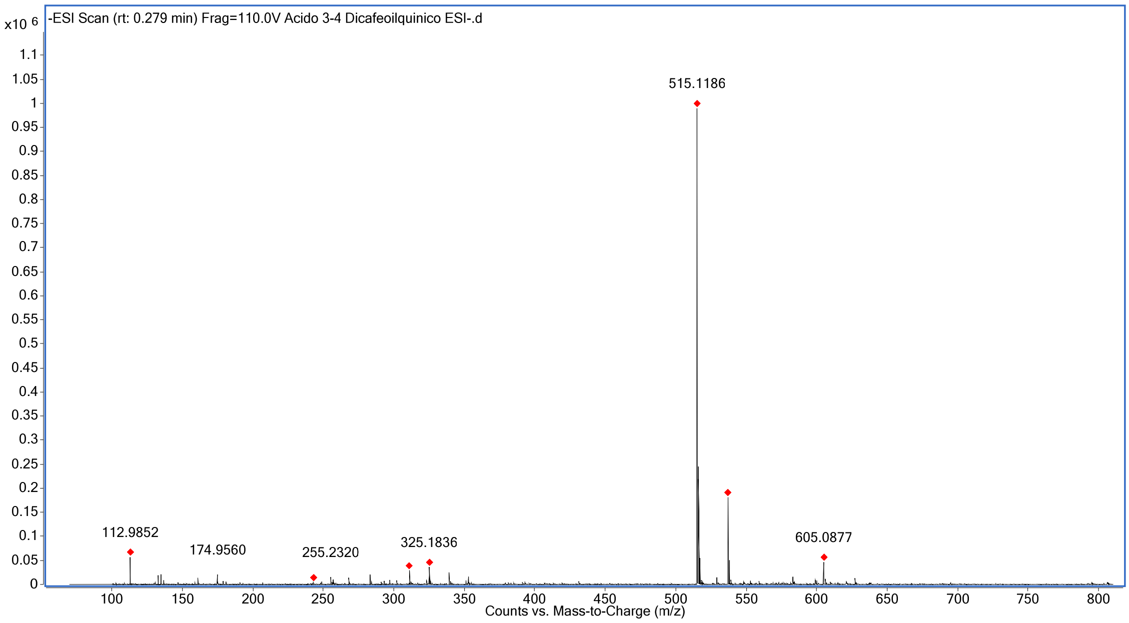

Figure S3. Full-scan QTOF/HRMS mass spectrum of 2 (m/z=515.1186).

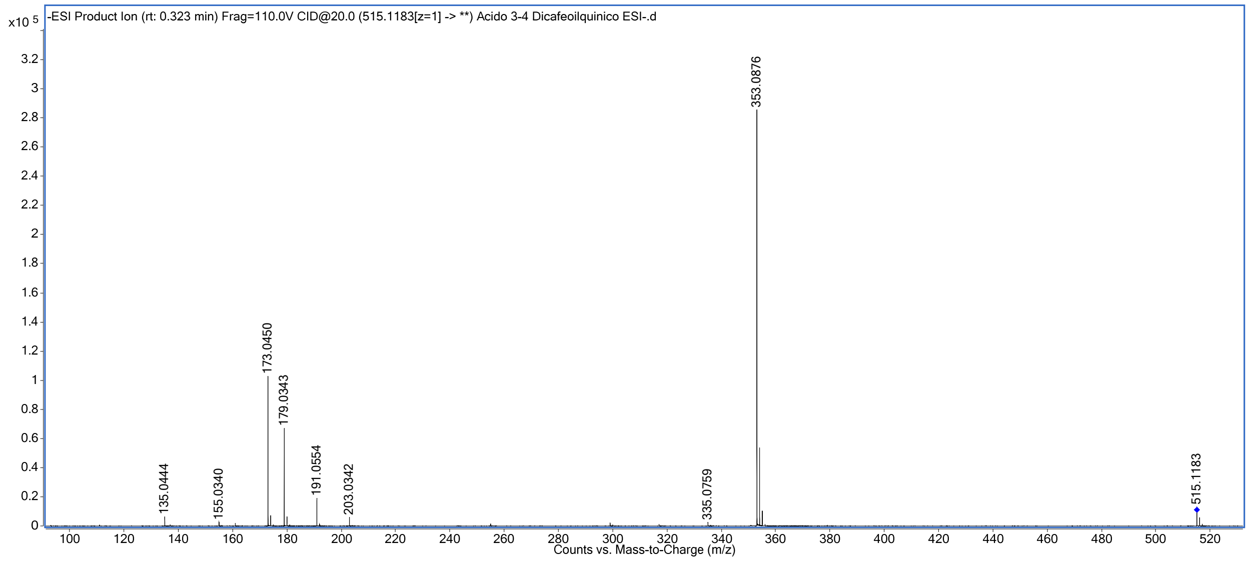

Figure S4. MS/MS fragmentation spectrum of compound 2 (m/z=515.1183).

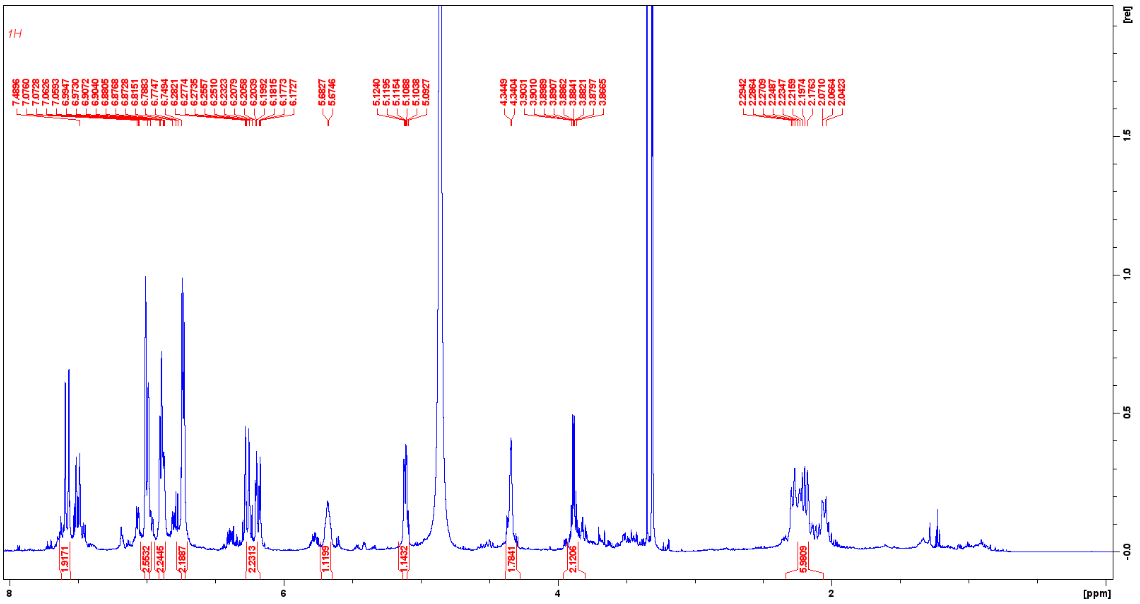

Figure S5. ^{1}H-NMR spectrum of compound **2** (600 MHz, CD_3OD).

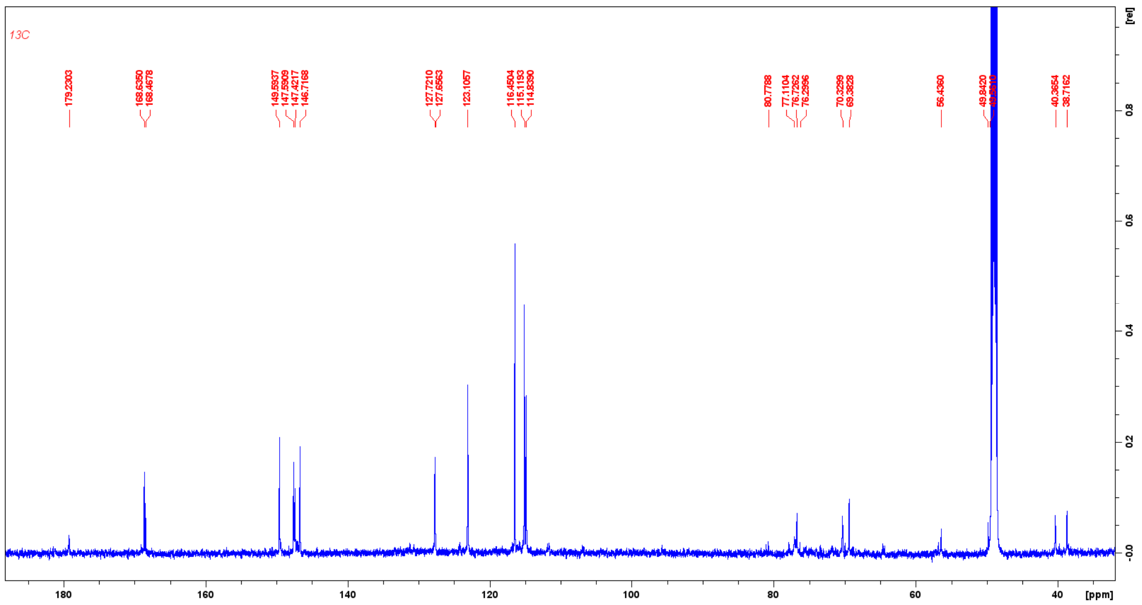

Figure S6. ^{13}C-NMR spectrum of compound **2** (150 MHz, CD_3OD).

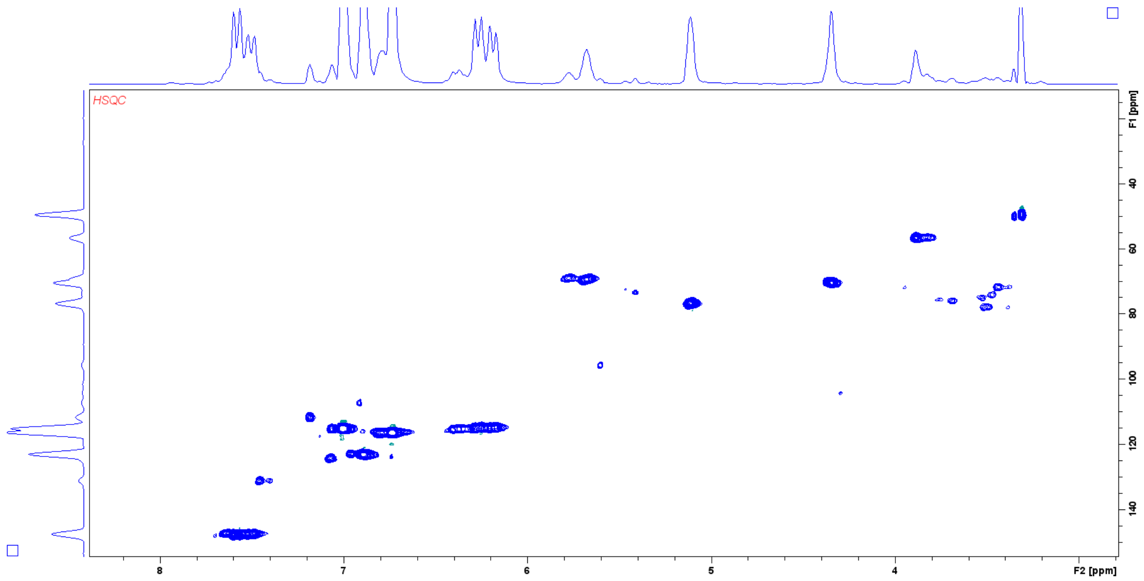

Figure S7. HSQC spectrum of compound **2** (150 MHz, CD_3OD).

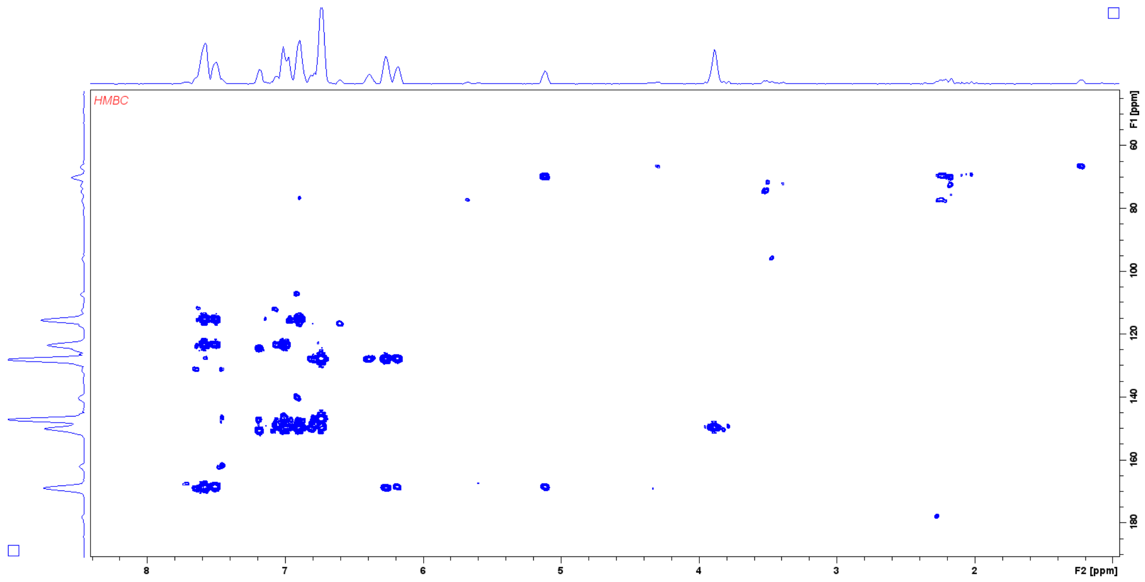

Figure S8. HMBC spectrum of compound **2** (150 MHz, CD_3OD).

Table S3. ^{1}H (600 MHz) and ^{13}C (150 MHz) NMR spectroscopic data of compound 2 recorded in CD_3OD.

	^{1}H (CD_3OD)	^{13}C (CD_3OD)	^{1}H (CD_3OD)*	^{13}C (CD_3OD)*
**1**		76.1	-	75.8
**2**	2.00-2.31*m*	38.4	2.00-2.40	39.4
**3**	5.68 (ddd; *J*=4.3; 3.7; 3.4)	69.0	5.62	69.4
**4**	5.11(dd; *J*=9.0; 3.4)	75.8	5.121	76.1
**5**	4.34 (ddd; *J*=10.3; 9.0; 4.3)	69.4	4.372	69.0
**6**	2.25 m	39.4	2.00-2.40	38.4
**7**		176.8		176.8
**1′**		127.8		127.7
**2′**	7.01 (dl)	115.3	7.01	115.2
**3′**		146.8		146.8
**4′**		149.7		149.7
**5′**	6.73 (d; *J*=8.0)	116.5	6.91	116.5
**6′**	6.9 (dl)	123.2	6.92	123.2
**7′**	7.58 (d; *J*= 14.4)	147.8	7.50	147.6
**8′**	6.26 (dd; 14.4)	114.8	6.29	114.7
**9′**		168.4		168.3
**1′′**		127.8		127.8
**2′′**	6.9 (dl)	115.3	7.02	115,3
**3′′**		146.8		146.8
**4′′**		149.7		149.7
**5′′**	6.73 (d; *J*=8.0)	116.5	6.74	116.5
**6′′**	6.89 (t; *J*=2.0)	123.2	6.92	123.2
**7′′**	7.58 (d; *J*=15.4)	147.8	7.60	147.8
**8′′**	6.26 (dd; *J*=14.4)	114.8	6.29	114.8
**9′′**		168.4		168.6

*Literature data (Tatefuji et al., 1996).

Table S4. ^{13}C NMR data of 1 and 1-labeled (CD_3OD) after incorporation of 1-^{13}C-D-glucose into root cultures.

			Relative intensity of signal (cm)		
			**L**	**U**	**ΔC (%)**
C	δ_c	δ_c*			
**1**	74.8	76.2	n	n	
**2**	36.8	38.2	0.8	0.2	**4.4**
**3**	70.0	71.3	n	n	
**4**	72.1	72.0	n	n	
**5**	70.5	73.5	n	n	
**6**	37.4	38.8	0.4	0.1	**4.4**
**7**	175.6	177.2	n	n	
**1'**	126.4	127.8	0.1	0.1	1.1
**2'**	113.8	116.5	1.7	0.3	**6.2**
**3'**	145.3	147.1	n	n	
**4'**	148.1	149.5	0.1	0.1	1.1
**5'**	115.1	115.3	0.3	0.3	1.1
**6'**	121.6	122.9	1.6	0.3	**5.9**
**7'**	145.7	146.8	1.5	0.4	**4.1**
**8'**	113.8	115.2	n	n	
**9'**	167.3	168.7	0.3	0.3	1.1

*Literature data (Liu et al., 2020).

U: control experiments with unlabeled precursor; L: labeling experiment with ^{13}C precursor; ΔC= 1.1% x L/U: increase in the relative intensity (significant increases in bold for enriched carbons). ^{n}-carbon signals of insufficient intensity for reliable measurement.

Table S5. ^{13}C NMR data of 2 and 2-labeled (CD_3OD) after incorporation of 1-^{13}C-D-glucose into root cultures.

			Relative intensity of signal (cm)		
			L	U	ΔC (%)
**C**	δ_c	δ_c*			
**1**	76.1	76.4	n	n	
**2**	38.4	38.6	0.4	0.1	**4.4**
**3**	69.0	70.1	n	n	
**4**	75.8	76.9	n	n	
**5**	69.4	69.3	n	n	
**6**	39.4	39.9	0.3	0.1	**3.3**
**7**	176.8	181.1	n	n	
**1'(1'')**	127.8	127.7	0.1	0.1	1.1
**2'(2'')**	115.3	115.1	1.6	0.3	**5.9**
**3'(3'')**	146.8	146.3	n	n	
**4'(4'')**	149.7	149.6	0.1	0.1	1.1
**5'(5'')**	116.5	115.1	0.3	0.3	1.1
**6'(6'')**	123.2	123.1	1.4	0.3	**5.1**
**7'(7'')**	147.8	147.6	1.0	0.2	**5.5**
**8'(8'')**	114.8	114.9	0.2	0.2	1.1
**9'(9'')**	168.6	168.4	0.1	0.1	1.1

*Literature data (Tatefuji et al., 1996).

U: control experiments with unlabeled precursor; L: labeling experiment with ^{13}C precursor; ΔC= 1.1% x L/U: increase in the relative intensity (significant increases in bold for enriched carbons). ^{n}-carbon signals of insufficient intensity for reliable measurement.

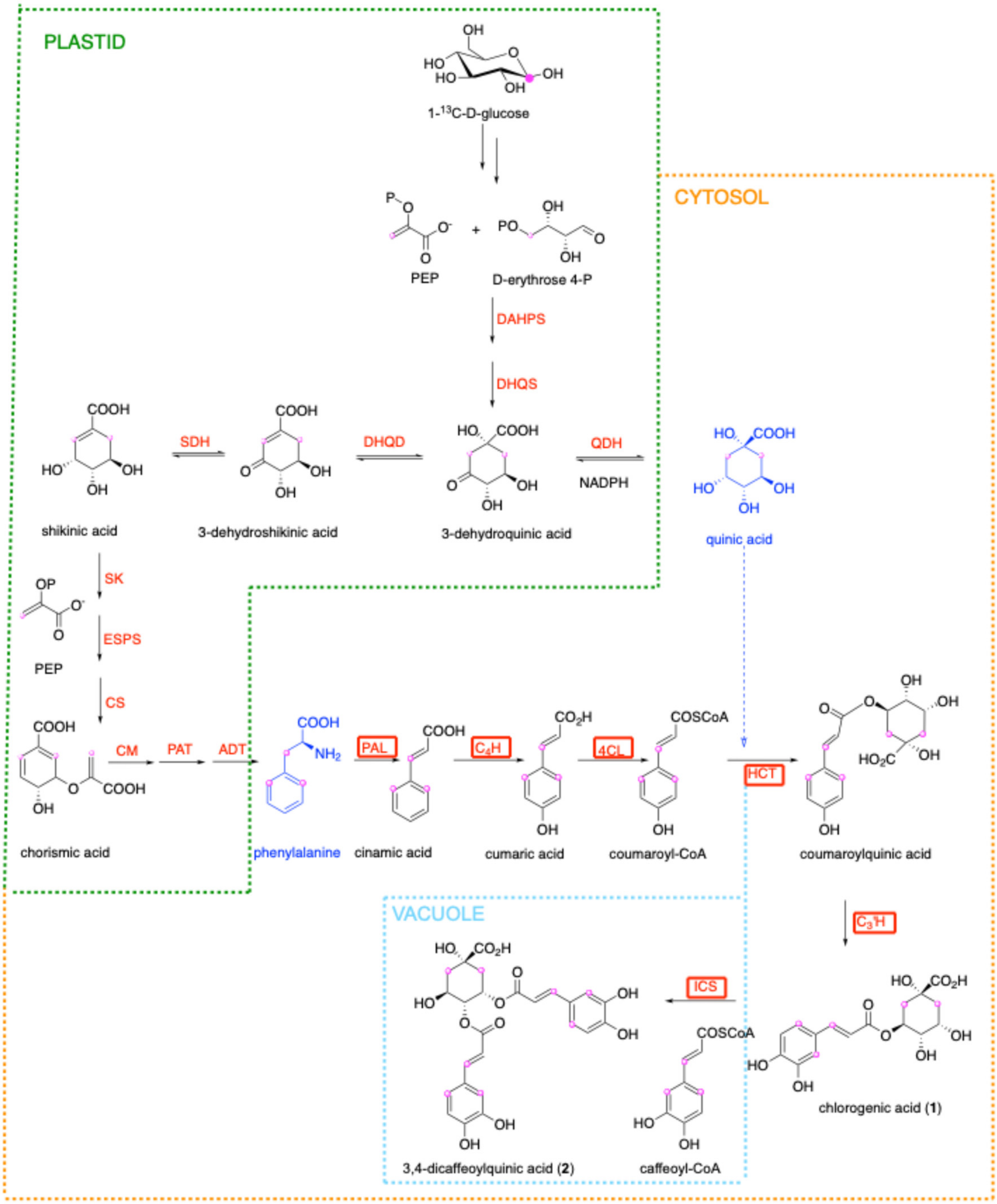

Figure S9. Biosynthesis of caffeoylquinic acids (compounds 1 and 2).

Table S6. Analytical validation parameters of the HPLC-DAD method for the quantification of compound 2.

Validation parameter	Evaluation	Result
**Linearity**	Regression equation	y = 49,227x + 64,307
	R^2	0.9995
	R	0.9997
**Precision**	Repeatability (RSD)	0.39–4.38%
**Accuracy / recovery**	Low level	96.97%
	Medium level	95.05%
	High level	99.41%
**Limit of detection**	LOD	1.0608 $\mu g.mL^{-1}$
**Limit of quantification**	LOQ	3.2146 $\mu g.mL^{-1}$

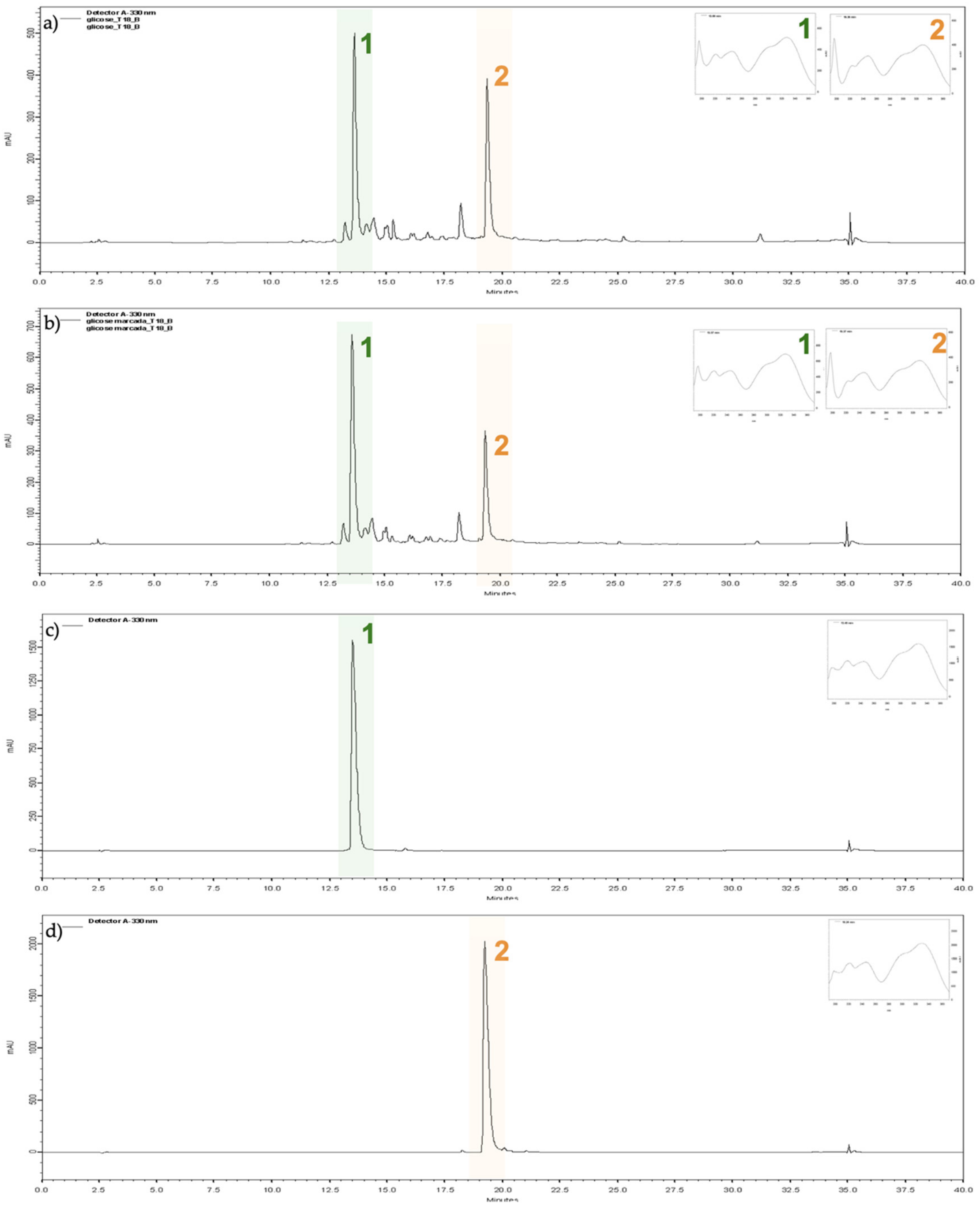

Figure S10. Chromatographic profiles of *U. tomentosa* root extracts obtained from: (a) the control experiment; (b) the isotope-labeling experiment; (c) chlorogenic acid (1) and (d) 3,4-di-O-caffeoylquinic acid (2) standards.

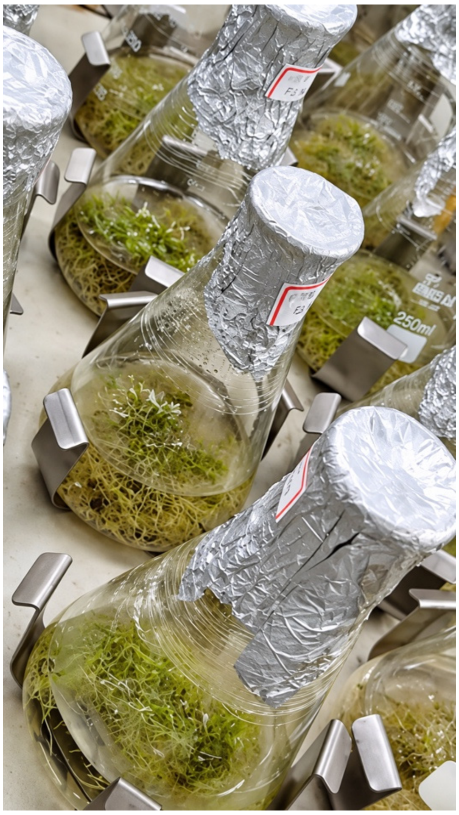

Figure S11. *U. tomentosa* root cultures.

References

Clifford, M.N.; Johnston, K.L.; Knight, S.; Kuhnert N. Hierarchical scheme for LC-MSn identification of chlorogenic acids. J Agric Food Chem. 2003, 51, 10, 2900-11.

Liu, W.; Nisar, M.F.; Wan, C. Characterization of phenolic constituents from *Prunus cerasifera* Ldb leaves. J. Chem. 2020, 2020, 5976090.

Tatefuji, T.; Izumi, N.; Ohta, T.; Arai, S.; Ikeda, M.; Kurimoto, M. Isolation and identification of compounds from Brazilian propolis which enhance macrophage spreading and mobility. Biol. Pharm. Bull. 1996, 19, 966–970.
